# Endoscopic outcomes in patients with AERD treated with topical antibiotics and intranasal corticosteroids

**DOI:** 10.3389/fcimb.2022.812215

**Published:** 2022-07-22

**Authors:** Jhon F. Martinez-Paredes, Garret Choby, Michael Marino, Devyani Lal, Osarenoma Olomu, Razan Alfakir, Janalee K. Stokken, Erin O’Brien, Angela M. Donaldson

**Affiliations:** ^1^ Department of Otolaryngology-Head and Neck Surgery, Mayo Clinic in Florida, Jacksonville, FL, United States; ^2^ Department of Surgery, University of Texas Rio Grande Valley, Edinburg, TX, United States; ^3^ Department of Otolaryngology-Head and Neck Surgery, Mayo Clinic in Rochester, Rochester, MN, United States; ^4^ Department of Otolaryngology-Head and Neck Surgery, Mayo Clinic in Arizona, Phoenix, AZ, United States; ^5^ Department of Speech-Language & Hearing Sciences, Auburn University, Auburn, AL, United States

**Keywords:** topical therapy for chronic rhinosinusitis, AERD, sinonasal rinses, culture, clinical outcomes, endoscopic sinus surgery (ESS), chronic sinusitis with nasal polyps

## Abstract

**Background:**

Identifying effective therapy for recalcitrant chronic rhinosinusitis with nasal polyposis (CRSwNP) is a major challenge; and subtypes such as aspirin-exacerbated respiratory disease (AERD) are even more difficult to treat. Evidence on topical antibiotics use in (CRSwNP) is lacking. Current consensus guidelines recommend against its routine use, but recent reviews show some benefit when managing recalcitrant disease after endoscopic sinus surgery (ESS).

**Objective:**

Evaluate the effect of culture-directed topical antibiotics on sinonasal outcomes in AERD patients with a positive perioperative sinonasal bacterial culture who have undergone ESS.

**Methods:**

A retrospective cohort study of AERD patients with positive sinonasal culture, who underwent ESS from 2016 to 2021 was performed. Forty-four patients were identified and stratified based on their postoperative medical treatment. Twenty-six underwent postoperative intranasal corticosteroids (INCS) alone, while eighteen underwent INCS plus a 4-weeks treatment with topical antibiotics. SNOT-22 and Lund-Kennedy score (LKS) were assessed preoperatively and at 4-weeks and 4-6 months after ESS.

**Results:**

A statistically significant improvement in the 4-weeks and 4-6 months postoperative SNOT-22 and LKS were noted within both groups (p<0.05). However, only a statistically significant difference was found in the 4-weeks postoperative LKS when comparing between treatment groups *(p=0.01)*. Our linear regression model demonstrated a relationship between the use of combined therapy with INCS and topical antibiotics and the LKS 4-weeks post ESS (p=0.015).

**Conclusion:**

In AERD patients with a confirmed sinus infection, the combination of culture-directed topical antibiotics and intranasal corticosteroid irrigations in the postoperative period can provide a short-term improvement in endoscopic scores.

## Introduction

Aspirin exacerbated respiratory disease (AERD) is a chronic inflammatory condition of the upper respiratory system (Samter and Beers, 1967; Samter and Beers, 1968). The pathophysiology of this condition involves an increase in the number of inflammatory cells and dysregulation in the synthesis of eicosanoids, leukotrienes, and prostaglandins (Laidlaw and Boyce, 2013). Clinically, AERD is associated with a high rate of nasal polyps recurrence and recalcitrant symptoms after endoscopic sinus surgery (ESS) (Walgama and Hwang, 2017).

Recalcitrant chronic rhinosinusitis (CRS) has been associated with the persistence of bacterial infections (biofilms), bacterial superantigens, immunodeficiency, and allergens, among others. While we currently do not have a clear understanding of how these factors affect the pathogenesis of CRS, there is evidence that persistent bacterial infections, such as those caused by Staphylococcus aureus (S. aureus), have an effect on the innate immune system. This immune response leads to an increase in the production of interleukin (IL)-4, IL-5, and IL-13, with a subsequent increase in immunoglobulin-E (IgE) and eosinophilia (Vickery et al., 2019). AERD patients have an increased level of antistaphylococcal enterotoxin IgE compared to those patients with aspirin tolerant chronic rhinosinusitis with nasal polyps (CRSwNP). This increased production of IgE leads to worsening eosinophil degradation and potential worsening effect on endoscopy and symptom scores (Vickery et al., 2019). We believe that the combined proinflammatory properties of bacterial infections and AERD make this a unique and important population to study.

Several studies have shown that the presence of bacterial biofilms has a negative impact on the healing process after ESS leading to persistence of postoperative symptoms and mucosal inflammation (Bendouah et al., 2006; Psaltis et al., 2008; Singhal et al., 2011). A recent study looking at the contribution of microbes in early disease recurrence after ESS found that perioperative positive culture with S. aureus was associated with poor outcomes, and that clearance of the bacteria led to 75% remission at 4-months after surgery (Maniakas et al., 2020). Focusing therapy on clearing perioperative infections in the initial postoperative period may alter the deleterious impact that bacterial pathogens have on the regeneration of epithelium and repopulation of the microbiome. Targeted therapy with topical antibiotics offers several benefits, including the reduction of possible systemic side effects and potential direct treatment of biofilms (Harvey and Schlosser, 2009).

Although topical antibiotics have been considered helpful in some settings of recalcitrant disease, its use is currently not recommended by the most recent International Consensus Statement on Allery and Rhinology (ICAR) on CRS and the European Position Paper on Rhinosinusitis and Nasal Polyps 2020 (EPOS2020) (Singhal et al., 2010; Singhal et al., 2011; Rudmik et al., 2013; Orlandi et al., 2016; Kim and Kwon, 2016). The studies considered in the recommendation did not specifically look at patients with CRSwNP or any of its subtypes. Specifically, of the current studies evaluating the use of topical antibiotics, none have explicitly looked at AERD patients (Sykes et al., 1986; Desrosiers and Salas-Prato, 2001; Videler et al., 2008; Shikani et al., 2013; Bonfils et al., 2015); therefore, we hope to add to the current body of literature by investigating the effect of this therapy in one of the most challenging patient populations to treat. We aim to evaluate the postoperative sinonasal outcomes in culture-positive AERD patients who received a combination of topical antibiotic and steroid irrigation versus those who received topical steroid irrigation alone.

## Methods

### Patients

This study was approved by the Institutional Review Board (IRB 19-009794). Patients diagnosed with AERD who underwent ESS from 2016 to 2021 and had a positive sinonasal culture taken preoperatively or intraoperatively, were retrospectively identified. Patients underwent sinonasal bacterial culture due to severity of symptoms and per surgeon’s discretion. Patients were analyzed based on whether they used topical antibiotics in the immediate postoperative period or not. One group received postoperative intranasal corticosteroids (INCS) only (Budesonide respule 0.5mg/2ml in 240ml saline, B.I.D), while the other group included patients who received combined therapy with INCS (Budesonide respule 0.5mg/2ml in 240ml saline, B.I.D) and topical antibiotic irrigation. Topical antibiotics were prescribed twice daily for a 4-weeks period. The type of topical antibiotic prescribed was based on culture and susceptibility results and included tobramycin (20mg capsule/240ml saline), mupirocin (2% ointment), and gentamicin (20mg capsule/240ml saline).

Patient records were reviewed for the following clinical characteristics: demographics, surgical history, and comorbidities. Results of sinonasal cultures and sinonasal outcomes were collected and placed in the RedCap project.

### Sinonasal Outcomes

The 22-item sinonasal outcome test (SNOT-22) questionnaire assesses patients’ symptoms with 22 different questions involving five different domains: rhinologic symptoms, extranasal rhinologic symptoms, ear/facial symptoms, sleep function, and psychological function (DeConde et al., 2014). The Lund-Kennedy endoscopic score (LKS) measures five endoscopic characteristics, including the presence of polyps, mucosal edema, sinonasal secretions, scarring, and crusting (Lund and Kennedy, 1997). Patients were asked to fill out the SNOT-22 questionnaire in the preoperative visit and at each postoperative follow-up. Additionally, the LKS was calculated at the preoperative and each postoperative visit according to the endoscopic findings. Data from the preoperative visit, 4-weeks, and 4-6 months postoperative were collected and analyzed for the purposes of this study. The previously reported minimal clinically important difference (MCID) for SNOT-22 of 8.9 points was used to evaluate clinically significant improvement over time (Chowdhury et al., 2017).

### Statistical Analysis

Statistical analysis was performed on SPSS software (version 25.0, IBM. Corp, Armonk, NY). Standard descriptive statistics were obtained and presented as percentages, mean ± standard deviations (SD). After confirming the normal distribution of the data, a Student’s t-test was performed to compare the means of each sinonasal outcome over time within and between groups. Additionally, a linear regression model was used with preoperative SNOT-22 and LKS scores as fixed factors in the analysis and aimed to evaluate: 1) The effect of postoperative topical antibiotics on sinonasal outcomes. 2.) The effect of concurrent oral antibiotics regimen during the topical antibiotic therapy on both sinonasal outcomes. A *p-value* <.05 was considered statistically significant.

## Results

### Patients

A total of 44 patients met the criteria for this study. The mean age among the overall cohort was 53 ( ± 14) with a 1.2:1 female/male ratio. Twenty-six patients received postoperative INCS only, while eighteen received combined therapy with INCS and topical antibiotics. Twenty-eight patients (63.6%) had a history of previous ESS. No differences were observed between the groups regarding gender, age, history of asthma, allergic rhinitis, smoking, and culture results (p>0.05). S. aureus was the most isolated pathogen, followed by Pseudomonas. When collecting the clinical information, we noted that all patients from the INCS only group received an empiric oral antibiotic regimen for two weeks as part of the postoperative treatment. In comparison, only six patients from the INCS and topical antibiotics group received a concurrent culture-directed oral antibiotic regimen. Prescribed oral antibiotic agents included levofloxacin, doxycycline, and trimethoprim/sulfamethoxazole (**Table 1**).

**Table 1:** Demographic and Clinical Data of the Study Population.

Characteristics	n = 44
Gender, No. (%), female	26 (59.1)
Mean age, y (SD)	53 (14)
Isolated Bacteria	
S*taphylococcus aureus*, No, (%)	19 (43.2)
*Pseudomonas aeruginosa*, No, (%)	7 (15.9)
Other pathogens	
- Propionibacterium acnes, No, (%)	4 (9.1)
- Streptococcus pneumoniae, No, (%)	4 (9.1)
- Haemophilus influenza, No, (%)	3 (6.8)
- Coagulase-negative staphylococci, No, (%)	3 (6.8)
- Prevotella	2 (4.5)
- Serratia rubidaea, No, (%)	1 (2.3)
- Enterobacter Aerogenes, No, (%)	1 (2.3)
Topical antibiotic agent	
- Tobramycin, No, (%)	14 (77.7)
- Mupirocin, No, (%)	3 (16.7)
- Gentamicin, No, (%)	1 (5.6)
Comorbidities	
Asthma, No. (%)	37 (84.1)
Allergic Rhinitis, No. (%)	23 (52.3)
Obesity, No. (%)	18 (40.9)
Smoking, No. (%)	12 (27.2)

### Sinonasal Outcomes

**Figure 1:**
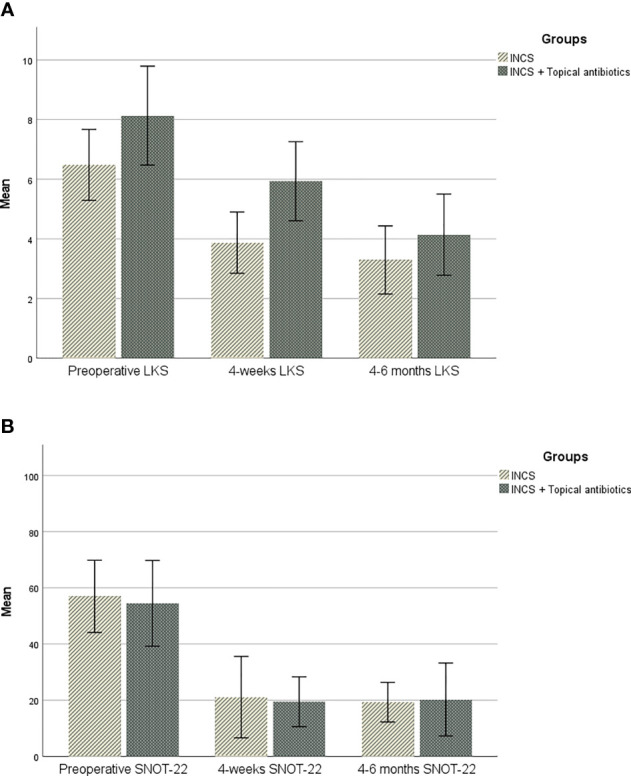
**(A)**. Change over time in sinonasal outcome test (SNOT-22). **(B)**. Change over time in Lund-Kennedy endoscopic score (LKS).

As outlined in **Figure 1**, sinonasal outcomes were collected and compared over time and between groups. There was no statistically significant difference in mean SNOT-22 between the two groups at any point in the study. However, a statistically significant difference in the 4 weeks postoperative LKS was found between them, as the INCS only group had a lower LKS (p-value=0.01). (**Tables 2**, **3**).

**Table 2:** Changes over time on sinonasal outcome test (SNOT-22) in the control and intervention groups (mean, S.D.).

	SNOT-22		
	Intranasal corticosteroids (INCS) group	Intranasal corticosteroids (INCS) + topical antibiotic group	Comparison between groups (p-value)
Preoperative	57 ± 24.9	54.5 ± 25	0.79
4-weeks postoperative	21.1 ± 18.6^a^	19.5 ± 11.9^b^	0.84
4-6 months postoperative	19.3 ± 11.8^c^	20.3 ± 16.2^d^	0.89

a p-value between preoperative and 4-weeks postoperative in the INCS group = 0.006.

b p-value between preoperative and 4-weeks postoperative in the INCS + topical antibiotic group = 0.004.

c p-value between preoperative and 4-6 months postoperative in the INCS group = 0.001.

d p-value between preoperative and 4-6 months postoperative in the INCS + topical antibiotic group = 0.029.

**Table 3:** Changes over time on Lund-Kennedy endoscopic score (LKS) in the control and intervention groups (mean, S.D.).

	LKS		
	Intranasal corticosteroids (INCS) group	Intranasal corticosteroids (INCS) + topical antibiotic group	Comparison between groups (p-value)
Preoperative	6.5 ± 2.8	8.1 ± 3	0.10
4-weeks postoperative	4 ± 2^a^	6 ± 3^b^	0.01
4-6 months postoperative	3 ± 3^c^	4 ± 2^d^	0.32

a p-value between preoperative and 4-weeks postoperative in the INCS group =0.017.

b p-value between preoperative and 4-weeks postoperative in the INCS + topical antibiotic group = 0.046.

c p-value between preoperative and 4-6 months postoperative in the INCS group = 0.0001.

d p-value between preoperative and 4-6 months postoperative in the INCS + topical antibiotic group =0.01.

For the INCS only group, a significant difference was found between preoperative and 4-weeks postoperative mean SNOT-22 [57 vs 21.1, (*p=0.006*)], and between preoperative and 4-6 months postoperative mean SNOT-22 [57 vs 19.3, (*p=0.001*)]. The change in SNOT-22 at 4-weeks and 4-6 months met the MCID threshold. A statistically significant difference was found between the mean preoperative and the postoperative LKS at both 4-weeks [6.5 vs 4, (*p=0.017*)] and 4-6 months postoperative scores [6.5 vs 3, (*p=0.0001*)].

In the topical antibiotic and INCS group, the postoperative mean SNOT-22 showed a sequential improvement over time. The change in SNOT-22 was statistically significant between the preoperative visit and 4-week postoperative visit [54.5 vs 19.5, (*p=0.004*)], and between preoperative and 4-6 months postoperative mean SNOT-22 [54.5 vs 20.3, (*p=0.029*)]. The change in SNOT-22 at 4-weeks and 4-6 months met the MCID threshold. A statistically significant difference was found between the mean preoperative LKS at both 4-weeks [8.1 vs 6,(*p=0.046*)] and 4-6 months postoperative scores [8.1 vs 4, (*p=0.01*)].

### Linear Regression

When analyzing the impact of the postoperative use of topical antibiotics, our linear regression model found a positive effect on the 4-weeks postoperative LKS (*β=* 2.0 [CI 95%: 0.43 to 3.7] *p=0.015).* However, it failed to demonstrate a detectable effect on the 4-weeks or 4-6 months postoperative SNOT-22 (*p=0.84*, and *p=0.88)*, and on the 4-6 months postoperative LKS (*p=0.32)* (**Table 4**). Regarding the impact of concurrent therapy with oral antibiotic regimens in a portion of our patients, our linear regression model did not show any effect on the 4-weeks postoperative SNOT-22 (*β=*-8.7 [CI95%: -26 to 8.5] *p=0.30)* and 4-6 months postoperative SNOT-22 (*β=*11.9 [CI 95%:-16 to 40] *p=0.37).* Additionally, no statistically significant effect on the 4-weeks postoperative LKS (*β=-*0.25 [CI95%: -0.84 to 0.32] *p=0.37)* and the 4-6 months postoperative LKS (*β=-*0.002 [CI95%: -1.0 to 1.0] *p=0.99)* was found.

**Table 4:** Linear regression analysis of the effect of postoperative topical antibiotics on sinonasal outcomes over time.

	β *value*	Standard Error	(95% CI)	*p*-value
*4-weeks after treatment*				
**SNOT-22** Postoperative topical antibiotic therapy
	-1.6	7.9	(-18.8 to 15.5)	0.84
LKS* Postoperative topical antibiotic therapy
	2.0	0.79	(0.43 to 3.7)	0.015
*4-6 months after treatment*				
**SNOT-22** Postoperative topical antibiotic therapy
	0.9	6.4	(-12.6 to 14.6)	0.88
LKS* Postoperative topical antibiotic therapy
	0.85	0.84	(-0.88 to 2.5)	0.32

LKS, Lund-Kennedy endoscopic score; SNOT-22, Sinonasal outcome test.

## Discussion

There is limited information about the effects of topical antibiotic irrigations in patients with CRSwNP. There is even less data on the effect of this medication therapy in patients with AERD. To our knowledge, this is the first study to look at the effect of topical antibiotics, specifically in the subset of patients with AERD and perioperative positive sinonasal cultures. The results of this study indicate that in AERD patients, the addition of culture-directed topical antibiotic irrigation in the immediate postoperative period leads to a statistically significant short-term improvement in LKS compared to those treated with nasal steroid irrigation alone. However, our study did not find a significant improvement in patient-reported symptoms measured by SNOT-22 when topical antibiotics were added to the postoperative treatment regimen compared to INCS alone.

The use of topical antibiotics in CRS remains controversial. Clinicians continue to use topical antibiotics in patients with recalcitrant sinus disease, and based on our findings, there is a potential benefit with combined therapy for those patients with a high risk for recalcitrant disease, including those with AERD. However, the most recent ICAR statement on rhinosinusitis and EPOS2020 have recommended against its routine use among CRSwNP patients (Orlandi et al., 2016; Fokkens et al., 2020a). Their recommendation was based on a few randomized controlled trials (RCTs), which each used a different drug delivery system and did not look specifically at AERD patients (Sykes et al., 1986; Desrosiers and Salas-Prato, 2001; Videler et al., 2008; Shikani et al., 2013; Bonfils et al., 2015). Only one RCT has analyzed the effect of topical antibiotic irrigations post-ESS (Jervis-Bardy et al., 2012; Orlandi et al., 2016; Fokkens et al., 2020a). They evaluated 25 patients with a documented S. aureus infection who received mupirocin nasal irrigations or saline irrigations for one month. Although they reported no significant changes in the SNOT-22 over time, they did note an immediate change of 4 points in the LKS after completing topical mupirocin rinses (Jervis-Bardy et al., 2012). This endoscopic improvement was not sustained after 2 to 6 months, however (Jervis-Bardy et al., 2012). Our results correlate with these findings as well as those from several retrospective studies (Orlandi et al., 2016; Lee and Davis, 2016). Our study does differ from previous studies, because we found that the improvement in LKS in the patients treated with topical antibiotics and INCS was sustained over a 6-month period of time. This observation was confirmed by the linear regression model which noted an association between the use of topical antibiotics and improved LKS at 4-weeks and 4-6 months after ESS. This may be explained by the concurrent and extended therapy with INCS after the topical antibiotics regimen was completed or the potential eradication of biofilms.

Bacterial biofilms have been associated with recalcitrant CRS (Singhal et al., 2010). Staphylococcus aureus and Pseudomonas are the most commonly isolated bacteria in patients with CRS. They are also considered biofilm-producer microorganisms, which can evade both the innate and adaptive immune response and exacerbate local inflammation responses (Kingdom and Swain, 2004; Bendouah et al., 2006; Zhang et al., 2015). Additionally, they are associated with recurrent infection after surgical or medical therapy due to their ability to elude standard antibiotic therapy and release bacteria in a planktonic form (Jervis-Bardy et al., 2011; Boase et al., 2013). A small number of animal and *in-vitro* studies have looked at the effect of topical antibiotics on S. aureus and Pseudomonas biofilms. Many of these studies found a significant reduction in biofilm surface area, with 90% eradication reported in one study (Chiu et al., 2007; Ha et al., 2008; Le et al., 2008).

Several studies looking at the effect of topical antibiotics included patients who received concurrent oral antibiotics (Jervis-Bardy et al., 2012; Ezzat et al., 2015; Shikani et al., 2018). These studies did not find an impact with the use of oral antibiotics. Given the potential for confounding results, we wanted to confirm that the use of concurrent oral antibiotics did not affect our study. Our linear regression model found that the use of a concurrent oral antibiotic regimen in those patients with prescribed topical antibiotic therapy had no effect on short-term and long-term sinonasal outcomes. The lack of effect by the addition of oral antibiotics is expected as previous studies looking at the use of oral antibiotics alone for acute rhinosinusitis only showed a slight benefit over placebo (Orlandi et al., 2016; Fokkens et al., 2020b).

As a retrospective study, there are several intrinsic limitations to consider. For example, limited data was available in some medical records, including previous medical history, preoperative and postoperative SNOT-22 scores, adverse effects of topical medication, and culturing method. Both groups in our study met the previously established MCID for SNOT-22 but it is difficult to discern how much surgery alone influenced the symptom change (Hopkins et al., 2009; Chowdhury et al., 2017). Additionally, the use of oral antibiotic therapy could confound the results of our study. We did perform a linear regression to evaluate its impact among the INCS and topical antibiotic group but were unable to complete this analysis within the INCS only group because all patients received an oral antibiotic regimen postoperatively.

This is a retrospective review focused on the results of medical intervention. Based on the construct of the study, we must consider that results may be altered by the lack of patient compliance, which was not documented in the chart. Additionally, because of the retrospective nature of our study, a large number of patients without complete SNOT-22 and LKS data had to be excluded. We are aware that our limited number of patients may have obscured a difference in the SNOT-22 outcomes; further studies with a larger sample will lead to a greater statistical power to confirm our findings. Given that our study looked at a unique population of patients, those with AERD, further studies to examine if these results are similar in non-AERD patients with nasal polyps are needed. There are limitations in assessing the results of the outcome variables due to the potential for observer bias in reporting the endoscopic LKS and confounding results related to the known benefits of ESS and INCS on symptom scores. Finally, longer follow-up could help to evaluate the recurrence of nasal polyps and sinus symptoms, as well as sustained long-term improvement in endoscopic findings.

## Conclusion

Our study suggests that in AERD patients with a positive perioperative bacterial infection, the addition of topical antibiotics irrigation to maintenance topical corticosteroids irrigation in the postoperative period provides short improvement in endoscopic scores. However, combination topical therapy has no effect on patient-reported postoperative SNOT-22, showing discordance between observed objective and patient-reported subjective findings.

## Data Availability Statement

The raw data supporting the conclusions of this article will be made available by the authors, without undue reservation.

## Ethics Statement

The studies involving human participants were reviewed and approved by Mayo Clinic Institutional Review Board. Written informed consent for participation was not required for this study in accordance with the national legislation and the institutional requirements.

## Author Contributions

JM-P: Data collection, statistical analysis and interpretation, manuscript writing, and editing. GC: Study design, manuscript writing, and editing. MM: Study design, manuscript writing, and editing. DL: Study design, manuscript writing, and editing. OO: Study design, manuscript writing, and editing. RA: Statistical analysis, manuscript writing. JS: Study design, manuscript writing, and editing. EO’B: Study design, manuscript writing, and editing. AD: Study design and conceptualization, statistical analysis, manuscript writing, and editing. All authors contributed to the article and approved the submitted version.

## Conflict of Interest

The authors declare that the research was conducted in the absence of any commercial or financial relationships that could be construed as a potential conflict of interest.

## Publisher’s Note

All claims expressed in this article are solely those of the authors and do not necessarily represent those of their affiliated organizations, or those of the publisher, the editors and the reviewers. Any product that may be evaluated in this article, or claim that may be made by its manufacturer, is not guaranteed or endorsed by the publisher.
